# Unpacking Food Fermentation: Clinically Relevant Tools for Fermented Food Identification and Consumption

**DOI:** 10.1016/j.advnut.2025.100412

**Published:** 2025-03-21

**Authors:** Elisa B Caffrey, Dalia Perelman, Catherine P Ward, Erica D Sonnenburg, Christopher D Gardner, Justin L Sonnenburg

**Affiliations:** 1 Department of Microbiology and Immunology, Stanford University School of Medicine, Stanford, CA, United States; 2 Stanford Prevention Research Center, Department of Medicine, School of Medicine, Stanford University, Palo Alto, California, United States; 3 Chan Zuckerberg Biohub, San Francisco, CA, United States; 4 Center for Human Microbiome Studies, Stanford University School of Medicine, Stanford, CA, United States

**Keywords:** FAM, FDM, dietary recommendations, fermentation, fermented foods, metabolites, microbiome, postbiotics, prebiotics, probiotics

## Abstract

Fermented foods have been consumed for millennia, valued for their extended shelf life, distinctive sensory properties, and potential health benefits. Emerging research suggests that fermented food consumption may contribute to gut microbiome diversity, immune modulation, and metabolic regulation; however, mechanistic insights and clinical validation remain limited. This review synthesizes current scientific evidence on the microbial and metabolite composition of fermented foods, their proposed health effects, and safety considerations for vulnerable populations. Additionally, we highlight the need for standardized definitions, serving sizes, and regulatory frameworks to enhance consumer transparency and research reproducibility. By providing a structured overview of existing data and knowledge gaps, this review establishes a foundation for integrating fermented foods into dietary recommendations and guiding future research directions.

**Statement of Significance**

Although fermented foods have demonstrated benefits to human health, the gap between scientific research and marketing claims, including lack of regulatory standards in labeling, can be disorienting to consumers seeking these potential benefits. This review provides an updated perspective on the role of fermented foods in health, emphasizing clinically relevant tools, research opportunities, and labeling recommendations to guide their identification and use.

## Introduction

Food fermentation has been harnessed for millennia for food preservation, detoxification, sustainability, flavor, and cultural practices. Commonly recognized fermented foods and beverages include sourdough bread, beer, yogurt, sauerkraut, kimchi, and kefir, with the number of fermented foods produced globally estimated to be in the hundreds [1,2]. Although fermented foods have become recognized as potential mediators of human immune and metabolic health, along with a number of other health conditions [[3], [4], [5], [6]], mechanistic understanding of how fermented foods impact patient populations is largely lacking. Randomized clinical trials [6,7] have generally shown a benefit to fermented food consumption, but limited trial size and length and relatively few trials focused on specific foods or patient populations make generalized conclusions difficult [[6], [7], [8], [9], [10]]. At the same time, commercial availability of fermented products has increased [11,12], accompanied by marketing claims that might not reflect the current biomedical research (See Box 1: Defining “Gut Health”). Here, we offer an updated perspective on the role of fermented foods in health and present clinically relevant tools for the identification and use of fermented foods.

BOX 1 **Defining “Gut Health”**

**Table:** 

“Gut health” is a nebulous term used to describe “absence of disease” in the gastrointestinal tract [32]. Products promoting “gut health” as a marketing tactic may include added fiber or probiotics. However, this term has no regulatory, medical, or scientific definition and instead might be used colloquially to denote the absence of disease or the alleviation of problematic symptoms, such as those associated with irritable bowel syndrome (IBS). Similarly, terms like “gut-friendly” or “balanced gut” are used to suggest support for normal digestive function, but these claims are not clearly defined by regulatory frameworks or consistently supported by scientific research outcomes. As discussed further in the text, foods labeled with vague claims such as “gut-friendly” may not contain live microbes or probiotics at all or any ingredient consistent with the current scientific understanding of having a positive impact on the gut microbiome, intestinal regulation, or overall health. This issue, while relevant to consumers, is particularly important for healthcare providers (HCPs), who should be equipped with the knowledge to guide patients in selecting appropriate products for their needs.

Alt-text: Box 1

## Defining Fermented Foods

Fermented foods have been defined by the International Scientific Association for Probiotics and Prebiotics (ISAPP) as “foods made through desired microbial growth and enzymatic conversions of food components” [6]. Although the biochemical definition of fermentation refers to a metabolic process occurring in the absence of oxygen, food fermentation describes a desired microbial processing of food occurring either in the presence or absence of oxygen, depending on the type of fermentation. This intentional microbial activity contrasts with food spoilage, which is the result of undesired microbial growth that degrades food quality, produces off-flavors, and may create safety concerns.

**FIGURE 1:**
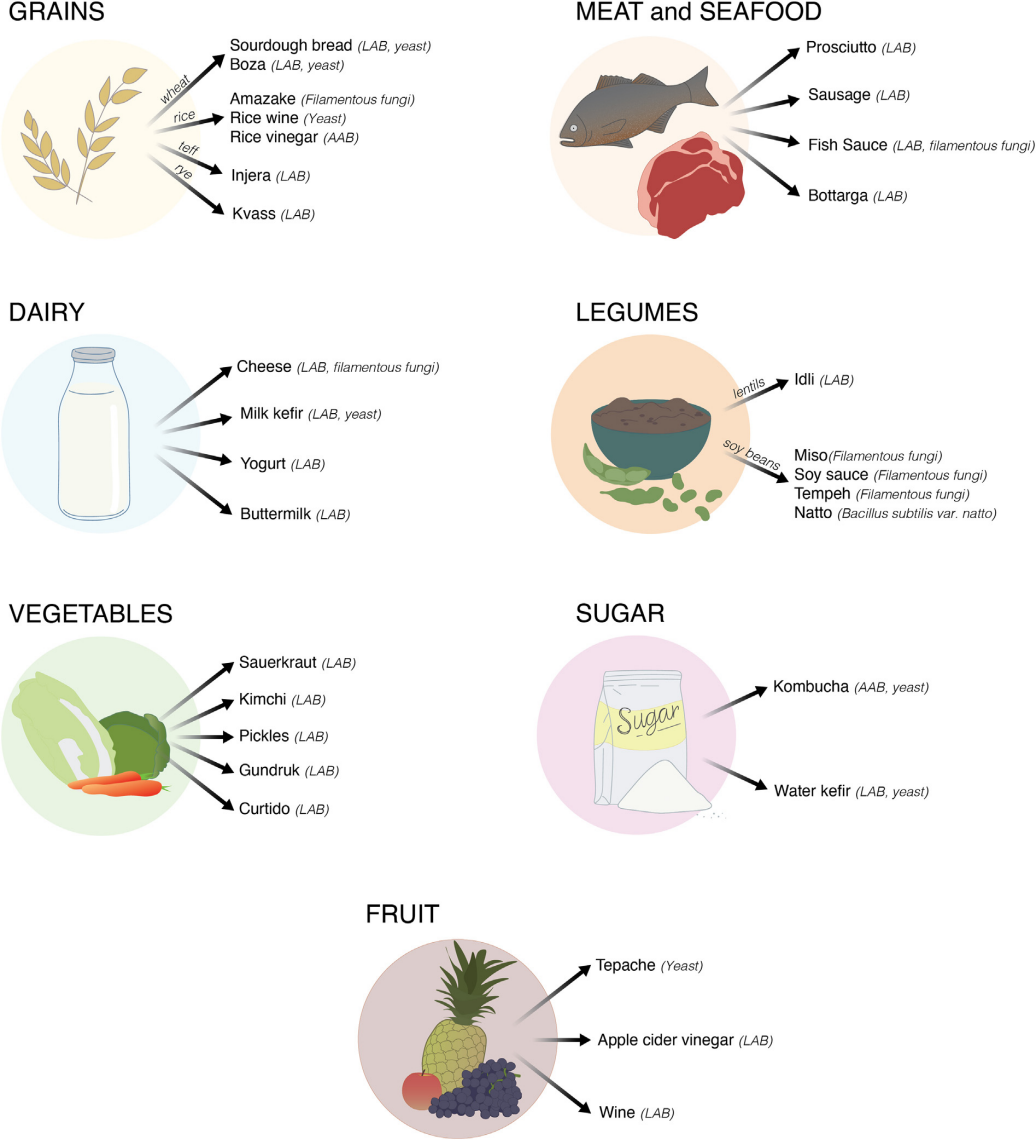
Broad categories of common fermented food by substrate, including microbial category primarily involved in fermentation. Although microbes that primarily drive the fermentation of each substrate have been identified, other microbes might also play a role in shaping the final safety, nutritional, and organoleptic properties of the final fermented product. LAB, Lactic acid bacteria; AAB, acetic acid bacteria.

The consumption of fermented food dates deep into human evolutionary history [13], supported by the enhanced ethanol metabolizing activity of alcohol dehydrogenase class IV (ADH4) enzyme 10 million years ago in our human ancestors [13]. The intentional practice of fermentation dates back to at least 14,000 BCE [14], with the diversity of available fermented foods driven by substrate availability and preparation methods [[15], [16], [17]]. Together, substrate and preparation shape which microbes will drive the desired fermentation process [18,19]. A variety of foods have been utilized in food fermentation production, including carbohydrate-rich grains, dairy, fruits and vegetables, protein-rich legumes, and protein- and fat-rich meat and seafood (Figure 1).

The microbes involved in the fermentation process (referred to as fermentation-associated microbes (FAMs)) vary based on a number of factors, including food substrate and assembly method [[18], [19], [20], [21]]. Use of spontaneous microbial communities (*de novo* or wild fermentation, as in sauerkraut) or an established community (starter, as in sourdough) fosters a diverse but poorly defined community. In other cases, a starter community with well-characterized strains can be used (as in yogurt). Community composition selection may vary based on a number of factors, including substrate and preparation style, which can differ significantly between commercial and at-home ferments. In the United States, there are no regulatory frameworks specifically designed for fermented foods. Instead, commercial preparation of fermented foods falls under general food safety regulations enforced by the Food and Drug Administration (FDA) and must comply with Good Manufacturing Practices and proper labeling requirements (FDA 21 CFR 117) [22]. One of the endpoints defined by the FDA for fermented food, specifically for food made with lactic acid- or acetic acid-producing microbes, is achieving a pH at or <4.6. This creates an inhospitable environment for foodborne pathogens such as *C. botulinum*, *L. monocytogenes*, *E. coli O157:H7*, and *S. flexneri* [[23], [24], [25], [26]]. Starter cultures used in commercial fermentation must also have Generally Recognized as Safe (GRAS) status or be approved as food additives (FDA 21 CFR 170).

In contrast, at-home fermentation typically relies more on spontaneous microbial communities or less-defined starter cultures, leading to reduced control over the microbial composition and final product. Despite this potential for variability, home fermentation is generally safe when proper guidelines are followed, as the naturally acidified environment and competitive microbial dynamics inhibit the growth of pathogens. Notably, there are no documented cases of foodborne illness associated with properly fermented foods, particularly vegetable-based ferments, in the United States [27,28]. Cottage food laws, which differ across states in the United States [[29], [30], [31]], allow individuals to prepare and sell certain fermented foods from their homes, provided they meet specific safety and labeling requirements. The differences in preparation style not only influence microbial communities but also contribute to the unique flavors, textures, and potential health benefits of fermented foods.

It is important to note that although fermented food may contain live and active cultures, it does not mean they meet the definition of probiotic as outlined by the WHO and FAO, which defines probiotics as “live microorganisms that when administered in adequate amounts, confer a health benefit on the host,” [33,34]. In the United States, the term probiotic has no legal definition and is considered primarily a dietary supplement by the FDA. However, with no legal definition and with the growing availability of probiotics on the market, the ISAPP has recently created a stricter definition. To meet ISAPP standards, probiotics should be taxonomically defined at a strain level, have an available genome sequence, should show evidence of a health benefit, and be alive and present at levels shown to provide a health benefit [35].

The history of probiotics and fermented food has long been intertwined. The microbiologist Stamen Grigorov first isolated a bacterial species called *Bulgarian bacillus* from Bulgarian “yahourth,” or curdled milk, in 1905 [36]. *B. bacillus* (now *Lactobacillus delbrueckii* subsp. *bulgaricus*) later became the focus of immunologist Metchnikoff [37], whose work propelled modern probiotic research. Today, probiotics have been used in a number of cases, including as therapeutics for pediatric acute gastroenteritis [38], and ongoing research is focused on improvement of current probiotics’ efficiency and effectiveness [[39], [40], [41], [42]]. In addition to probiotics that play a role in fermentation, as with *L. delbrueckii* subsp. *bulgaricus* in yogurt, other probiotics might be added to fermented food following fermentation and accompanied by a “probiotic” label. For example, the addition of a spore-forming probiotic species of *Bacillus*, such as *B. coagulans* or *B. subtilis,* are added as inert, living, but inactive microbes to a number of commercial foods. Although they are not capable of contributing to the fermentation process, the spores are able to withstand harsh environmental conditions, including high heat, desiccation, and chemical exposure, processes that make foods shelf-stable. Such foods can claim on their labels that it “contains live and active cultures” or “includes beneficial bacteria” without having undergone fermentation. Addition of commercial microbial strains added as probiotics, considered dietary supplements by the FDA, must be listed on the label (21 CFR § 101.4). If a fermented food does not list a strain, fermentation was likely initiated via microbes in the environment (e.g., sauerkraut) or through an established but uncharacterized microbial community (e.g., symbiotic culture of bacteria and yeast or sourdough starter).

During the fermentation process, microbes produce metabolites, small chemical products of metabolism, which can act as an energy source and signaling compounds [4,43]. Metabolites enriched in fermentation due to microbial metabolism are referred to as fermentation-derived metabolites (FDMs) and are distinct from metabolites already present in the substrate (e.g., polyphenols found in tea leaves); however, FDMs may be derived from microbial transformation of endogenous compounds. Primary FDMs, such as organic acids (e.g., lactic acid, acetic acid) or ethanol, are the dominant end products of fermentation and can accumulate in large quantities. Secondary metabolites such as terpenoids, a diverse group of compounds with a wide range of functions, including conferral of flavor [44,45], are produced later in the fermentation process and are typically found in smaller quantities [46]. These metabolites produced during the fermentation process may be desirable, playing a role in shaping microbial community development while enhancing the nutritional properties, safety, flavor, and texture of the food. The spectrum of metabolites produced during food fermentation defines many properties of the food, including organoleptic properties, and are increasingly associated with health impact [4,5,47].

Ethanol is a FDM produced by yeast fermentation of sugars, often intentionally through the use of native yeast or by the addition of commercial yeast (e.g., *Saccharomyces cerevisiae*) in wine and beer making. Recent guidance from The Lancet and Dietary Guidelines for Americans recommends against consumption of alcohol [48,49]. Given the great diversity of fermented foods available, alcoholic ferments should not be regarded as a substitute or counted toward the total daily intake of fermented foods. In addition, alcohol can be produced in any food fermented with yeast, including kombucha and kefir. Although levels of alcohol in commercial kombucha must be <0.5% alcohol to meet the “nonalcoholic” regulation, factors such as fluctuation in temperature during transportation and storage and pasteurization status may lead to slight fluctuations in alcohol abundance. For that reason, kombucha is not recommended for consumption during pregnancy [50]. Milk kefir can similarly contain a low abundance of alcohol, depending on the type of milk and storage method [51,52]. Given that the alcohol content of these foods is very low, they are generally included in the fermented foods category as healthy options for most people.

**FIGURE 2:**
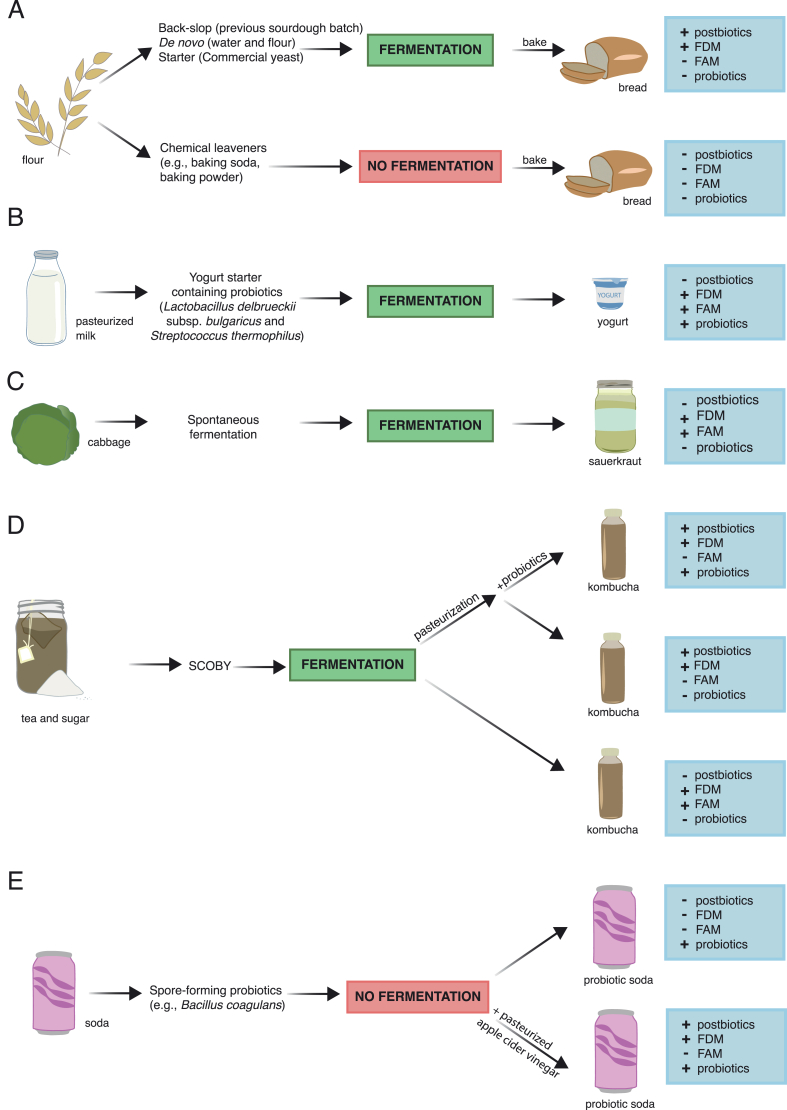
Food preparation impacts final postbiotic, metabolite, live microbe, and probiotic composition. Substrate, microbial assembly, and pasteurization can all impact the final profile of the food, including presence of live fermentation-associated microbes (FAMs), probiotics, fermentation-derived metabolites (FDMs), and postbiotics in the food. For example, in bread production, (A) addition of a sourdough starter (back-slop), production of a new batch of sourdough from wild yeast (*de novo*), or use of a commercial yeast starter will all lead to production of FDMs. Following the fermentation rise, baking of the bread will kill the active microbes, leaving the postbiotics and heat-stable FDMs. Use of a chemical leavener, however, will not result in a microbially fermented product, thus lacking any FDMs and postbiotics in the final bread. Yogurt production (B) will typically begin with pasteurized milk, followed by the addition of probiotic yogurt-producing strains. Following fermentation, yogurt is typically consumed with the live microbes; the strains used for fermentation may be probiotics, and in some cases, probiotic strains are added after fermentation. In sauerkraut production (C), as with many vegetable ferments, no starter is added, relying on the spontaneous microbial community to initiate fermentation. Unpasteurized vegetable ferments are typically indicated by a label including the terms “raw,” “wild,” or “living.” Sauerkraut usually does not contain probiotics because the specific strains from spontaneous fermentation are undefined. Kombucha (D) can be produced in a number of ways. First, the symbiotic culture of bacteria and yeast starter (an undefined microbial community) will be added to tea and sugar. Following fermentation, kombucha may be bottled and consumed without pasteurization, resulting in a product containing live microbes and metabolites but not probiotics. Alternatively, kombucha may be pasteurized, containing only postbiotics and FDMs. At any point in the process, probiotics may be added and labeled on the commercial bottle for sale. If no strains are listed, no probiotics have been added. Finally, there are cases in which probiotics are added, but fermentation has not taken place (E), like in the case of probiotic sodas, which are shelf-stable. Such products contain a probiotic but no FDMs or postbiotics. However, if a fermented ingredient is added, such as pasteurized apple cider vinegar, the final product would contain FDM and possibly postbiotics from the vinegar fermentation process, although, in this example, the final consumed concentration would be much lower compared with consuming a fermented food. It should be noted that whereas many ferments that contain live microbes do not have bonafide probiotics, they often contain strains that share properties with and are close relatives of known probiotics.

The relative importance of live microbes and FDMs in achieving health benefits remains an open area of investigation. Pasteurization, a highly controlled heat treatment of food to kill microbes [53], typically serves the following 2 purposes: *1*) eradication of potential pathogens and spoilage microorganisms prior to addition of a FAM or probiotic (in the case of yogurt made from pasteurized milk) or *2*) eradication of the FAM after fermentation to improve shelf stability of the final product (e.g., in the case of some kombuchas) (Figure 2). When fermented foods are pasteurized, consumers ingest both the inactivated microbial cells, any cell components degraded during pasteurization, and FDMs that were produced during fermentation and were not degraded during the pasteurization process [54,55]. Although there is no legal distinction, according to the ISAPP, these components should be classified as “postbiotics” or “inanimate microorganisms and/or their components that confer a health benefit on the host” [56,57]. FDMs, in the absence of inanimate microorganisms and/or their components, do not meet the criteria for postbiotics [[56], [57], [58]] (See Box 2: Glossary of Discussed Terms).

Box 2 **Glossary of Discussed Terms**

**Table:** 

**Fermented Foods:** Defined by the ISAPP as “Foods made through desired microbial growth and enzymatic conversions of food components” [6].
**Probiotics:** Defined by WHO/FAO as **“**Live microorganisms that, when administered in adequate amounts, confer a health benefit on the host” [33,34]. ISAPP has created a stricter definition: for a microbe to be considered probiotic, it must *1*) show evidence of a health benefit, *2*) be taxonomically defined, and *3*) the genome sequence must be available. In addition, for a food to be considered probiotic, the levels of the probiotic microorganism in the food must be “live and present in levels demonstrated to provide benefit” [35]**.**
**Fermentation-associated microbe(FAM):** Any microbe (including bacteria, yeast, and fungi) that is involved in the desired conversion of food components during fermentation. Microbes promoting food spoilage would not be included in this category.
**Metabolites:** Molecules produced during metabolism.
**Fermentation-derived metabolite(FDM):** Metabolite produced as a result of microbial activity during food fermentation.
**Postbiotic:** Defined by the ISAPP as a “preparation of inanimate microorganisms and/or their components that confers a health benefit on the host.” A postbiotic may include an intact but inactive microorganism or structural debris such as pili or cell wall components. Metabolites may be present but not required. Although loss of viability of probiotics and FAMs naturally occurs in fermented food products, postbiotics are distinct in that a step must be taken to deliberately inactive (e.g., through heat treatment or pasteurization) the living microbes.

Alt-text: Box 2

The health effects of consuming postbiotics compared with live probiotics, FAMs, and FDMs are still being researched. A critical question is whether pasteurized versions of fermented foods can provide the same, worse, or better health benefits as their unpasteurized counterparts. For example, one clinical trial comparing consumption of pasteurized and unpasteurized sauerkraut in patients with irritable bowel syndrome (IBS) showed a comparable relief from symptoms in both groups compared with the control group [59]. A second randomized crossover clinical trial comparing pasteurized and unpasteurized sauerkraut consumption in healthy participants found that consumption was well tolerated. However, serum short-chained fatty acids (SCFAs) concentrations increased significantly only in the group consuming pasteurized sauerkraut [60]. In mice, a study comparing sauerkraut brine to filter-sterile sauerkraut brine showed a significant increase in small intestinal regulatory T cells in both groups compared with control. Interestingly, the filter-sterile sauerkraut brine showed a slight increase in regulatory T cells, supporting the hypothesis that some fermented food consumption health benefits might be independent of living microbes [47]. With a growing interest in microbial postbiotics and FDMs for beneficial health outcomes, understanding the factors dictating postbiotics and FDM stability and mechanism of action will inform fermented food product design while maximizing health benefits for the consumer.

Fermented products can be largely divided into the following 3 categories, based on pasteurization status and probiotic inclusion: *1*) an unpasteurized fermented food that contains both live FAMs and FDMs; *2*) a pasteurized fermented food that contains both FDMs and inactive FAMs; and *3*) A pasteurized fermented food containing both FDMs and inactive FAMs, with added probiotics (Figure 2). In addition, a probiotic culture may be added to a nonfermented product, in which case the product would be probiotic but without FAMs or FDMs and would not be considered fermented. Pasteurization of fermented food is popular among manufacturers to increase shelf life of a product by killing fermenting microbes and arresting fermentation. By adding a viable microbe that does not ferment the food (e.g., *Bacillus* species, as discussed above), the packaging can claim the product contains “live” cultures. Additionally, although lactic and acetic acid can be produced during the fermentation process, addition of these compounds in order to recreate the taste of a fermented food (as listed on the ingredients) does not mean the food has undergone fermentation. Vegetables brined in vinegar (acetic acid to produce pickled vegetables) or sour cream thickened with lactic and/or citric acid are compatible with longer shelf stability due to the exclusion of living microbes. Although the difference in benefit between the 3 categories described above still needs to be explored, it is important to emphasize that inclusion of a probiotic strain or fermentation metabolite does not mean the food is fermented.

## A Clinical Review of Fermented Food Consumption

Recent research has led to an increased interest in understanding the health benefits of fermented food consumption. Although these have been extensively reviewed [[4], [5], [6], [7], [8],^61^], here we offer a brief summary.

During fermentation, production of organic acids, bacteriocins, and other antimicrobial compounds play an important role in enhancing food safety and inhibiting the growth of potential human pathogens. Safe food preservation was especially important prior to refrigeration, where fermentation of milk into cheese or cabbage into sauerkraut allows for much longer storage and calorie preservation than the unfermented, unrefrigerated food would allow. Fermented foods have also been studied for their enrichment in nutritional value, including vitamins C, B12, and K, riboflavin and folate. Antioxidants, such as polyphenols and flavonoids, increase following fermentation in foods such as kombucha [62]. Enrichment and depletion of these metabolites can impact flavor. The most significant in food fermentation are organic acids such as lactic acid and acetic acid, which contribute to the tang and brightness of ferments like kimchi and vinegar.

Clinical interest in fermented foods has looked at the impact of their consumption on both healthy and patient cohorts. Two large retrospective studies, one looking at fermented food consumption (*n* = 6811) [63] and the other looking at intake of live microbes and fermented food intake (*n* = 46,091) [64], both reported positive health findings associated with fermented food consumption, including shifts in gut microbiome composition. Similar findings were observed in a small randomized controlled trial, showing an increase in gut microbiome diversity and a decrease in markers of inflammation in healthy adults consuming fermented foods [3]. A number of clinical trials and retrospective studies have further supported a trend in decreases in inflammation, gastrointestinal symptoms, and risk of type 2 diabetes mellitus (Table 1) [3,10,59,60,[65], [66], [67], [68], [69], [70], [71], [72]]. An additional consideration is the role fermented foods provide as an alternative to ultraprocessed foods. Consuming fermented drinks, such as kombucha or water kefir, as an alternative to beverages such as classic sodas offers greater nutritional benefits compared with calorie-dense drinks with minimal nutritional value, which have become increasingly recognized for having a negative impact on human health [73].

**TABLE 1:** Summary of select recent human studies on fermented foods.

Study	Study type	Population	Participant size	Length of study	Serving	Fermented food	Key findings
Han et al., 2015 [65]	RCT	Overweight women	24	8 wk	60 g 3/d	Kimchi	Gut microbiome composition shift, change in immune metabolic pathway gene expression
Díaz-López et al., 2016 [66]	Prospective	T2DM	3454	4 y	1 serving/d	Yogurt	Decreased risk of T2DM
Korem et al., 2017 [67]	RCT	Healthy	20	2 wk	100–145 g/d	Sourdough bread	No significant differences noted in interpersonal variation of effect
Nielsen et al., 2018 [59]	RCT	IBS	34	6 wk	75 g	Sauerkraut	Reduced IBS symptoms with both pasteurized and unpasteurized sauerkraut, Gut microbiome composition shift
Chen et al., 2019 [68]	RCT	NAFLD, MetS	100	24 wk	220 g/d	Yogurt	Gut microbiome composition shift, significant T2DM biomarker improvement
Yılmaz et al., 2019 [69]	RCT	IBD	45	4 wk	400 mL/d	Kefir	Decreased inflammatory markers, symptom improvement
Wastyk/Fragiadakis et al., 2021 [3]	RCT	Healthy	36	17 wk	5–6 servings/d	Any	Gut microbiome composition shift and diversity increase, decrease in inflammatory markers
Kim et al., 2022 [70]	RCT	IBS	90	12 wk	210 g/d	Kimchi	Symptom alleviation, gut microbiome composition shift, immune benefit
Akamine et al., 2022 [71]	RCT	MetS	40	4 wk	350 g/d	Amazake	Increase in plasma SCFA, gut microbiome compositional shift
Bourrie et al., 2023 [72]	RCT	Healthy	21	12 wk	350 g/d	Kefir	Reduction in markers of inflammation dependent on type of kefir consumed
Ecklu-Mensah et al., 2024 [10]	RCT	Healthy	24	8 wk	16 oz/d	Kombucha	No significant differences, increase in kombucha-associated microbes during intervention
Schropp et al., 2025 [60]	RCT	Healthy	87	8 wk	100 g/d	Sauerkraut	No significant difference in gut microbiome diversity, increase in serum SCFA in pasteurized sauerkraut group

For ongoing studies, visit clinicaltrials.gov.

*Abbreviations*: IBS, irritable bowel syndrome; IBD, inflammatory bowel disease; MetS, metabolic syndrome; NAFLD, nonalcoholic fatty liver disease; RCT, randomized controlled trial; T2DM, type 2 diabetes mellitus.

Although consumption of fermented foods has broadly been associated with improved health outcomes (see section on “Defining Fermented Foods and A Clinical Review of Fermented Food Consumption”), commercial fermented product nutritional, microbial, and metabolite profiles may vary from products used in clinical studies. Factors such as production style, storage conditions, fermentation methods, and timing can significantly affect the FAM community and FDM concentrations. Recent work quantifying aryl-lactates in commercial fermented foods [74], known for their beneficial impact on immune function [[75], [76], [77]], has shown that similar fermented commercial products show differences in concentration, and concentration can continue to change during storage. Understanding how these variables influence the FAM and FDM profiles of fermented foods is critical for developing mechanistic insights into their impact on health. Although sampling and cost of testing can be a limiting factor in clinical work, future clinical trials involving fermented foods should adopt a standardized approach to reporting the microbial, nutritional, and molecular composition of the fermented product being consumed by participants, which will be critical to reproducibility and generalizability of findings.

Despite variations in the composition of fermented food products, health benefits have been observed across a number of different studies, suggesting that the consumption of fermented foods is generally advantageous despite the current lack of product standardization and limited mechanistic insight. Interestingly, health benefits are evident both when individuals consume a single type of fermented food [59,65,68,69,71,72,78,79] and when individuals consume a diverse array of fermented foods [3]. More work is needed to investigate these foods to understand the basis of these clinical benefits. Similar to commercially produced fermented foods and fermented foods prepared for clinical trials, there is limited information about how making fermented foods at home impacts FAM and FDM concentrations. To date, no clinical trials have looked at or specifically recommended consumption of at-home ferments during the trial period, highlighting a research need to better understand health implications.

Another consideration when conducting clinical trials and translating the clinical work includes defining serving size. Although the serving size varies across clinical trials, recent work has used Reference Amounts Customarily Consumed (21 CFR. 101.12(b)) as a tool to define fermented food serving size. However, there is a need for research to establish evidence-based serving sizes tailored to different fermented food types, patient populations, and health conditions. For example, foods such as sauerkraut, kimchi, and pickles, are typically made with 1.5%–2% salt by weight, whereas miso can contain upwards of 20% salt, clearly containing higher concentrations of sodium compared with their unfermented counterparts (e.g., kimchi compared with napa cabbage). Sodium intake is often a concern for individuals with hypertension or cardiovascular disease. However, research, including both animal and clinical studies, suggests that miso consumption does not necessarily lead to increases in blood pressure, possibly due to its unique composition and bioactive compounds [78,79]. In addition, a clinical trial asking participants to supplement their typical diet with fermented food showed no increase in sodium intake [3]. To balance the nutritional benefits of fermented foods, such as supporting the immune system and promoting microbiome diversity, with sodium intake, serving size recommendations need to be tailored to meet the needs of individuals, particularly those sensitive to sodium.

In addition to serving size, it is likely that consumption of fermented foods might be the largest load of live microbes and their FDMs that most consumers will be exposed to that day. Our current food supply is mostly devoid of microbes and vastly different than the microbe-laden diet humans evolved with. Although sanitation of food has reduced the prevalence of foodborne disease, the overall health impact of a relatively sterile food supply, as proposed by the hygiene hypothesis [80], remains unclear. Research suggests that fermented foods promote gut microbiome diversity, countering the diversity-depleting effects of the industrialized lifestyle [4,81,82]. A diverse microbiome is associated with better health in a variety of studies, and a more diverse microbiome has been shown to be more resistant to invasion and resilient to perturbations [81,[83], [84], [85]].

There is currently no established number of microorganisms that are recommended to ingest. A study estimating the microbial intake in the United States found that for the majority, the estimated microbial range consumed by children and adults was 10^4^–10^7^ colony forming units/g [86], primarily from fruits, vegetables, and fermented dairy. Dairy ferments such as yogurt contain an estimated 10^7^-10^9^ colony forming units/g, with concentrations highly variable depending on the product type [87] and often less than is stated on product packaging. There is a clear need to establish both the utility and recommended level of microbial intake for health benefit and to understand if there is a level of daily excess exposure [88].

## Special Health Considerations

Though fermented foods can convey health benefits for many individuals, there are several populations who should be wary of fermented food consumption, including immunocompromised patients, pregnant populations, patients suffering histamine intolerance, and patients with IBS or small intestinal bacterial overgrowth (SIBO). These populations should exercise caution when consuming fermented foods due to the potential for harmful bacterial contamination or overgrowth and follow the advice of medical professionals when selecting appropriate fermented foods for consumption. Continued research focused on FAM and FDM characterization and clinical trials addressing these patient populations will optimize benefits, inform safety risks, and allow for clearer clinical guidance on specific fermented food types.

### Fermented foods and immunocompromised patients

Immunocompromised individuals, who include those on immune-suppressing medications (such as cancer therapeutics and corticosteroids), organ transplant recipients, and individuals with compromised immune systems due to other conditions, are at an increased risk of foodborne infections due to their weakened immune systems. Certain fermented foods, which may contain live bacteria and fungi, can pose a risk of infection in these populations. The Centers for Disease Control and Prevention advises that people with weakened immune systems should avoid unpasteurized fermented products to reduce the risk of foodborne illness [89]. For example, fermented products made from raw dairy products are not recommended by the center for disease control and health professionals due to increased risk of exposure to *Campylobacter*, *Cryptosporidium*, *Escherichia coli*, *Listeria*, *Brucella*, and *Salmonella* [90]. Additionally, a 2018 study [91] highlights that mold used in traditional fermented foods, such as those from the *Mucor* and *Rhizopus* species, can cause invasive fungal infections in immunocompromised individuals. Therefore, it is crucial for immunocompromised patients to consult healthcare professionals before consuming fermented foods to ensure their safety.

### Fermented foods in pregnancy

Pregnant individuals fall into the category of those with altered immune systems due to the immune modulation that occurs during pregnancy. There is emerging research suggesting fermented foods during pregnancy convey health benefits for both mom and offspring [92]; however, it is still recommended to follow the guidance of a healthcare provider when selecting appropriate fermented foods for consumption.

### Fermented foods in histamine intolerance

Research suggests that histamine concentrations may be elevated in the mucosal lining of the intestines in individuals with Crohn's disease, a type of inflammatory bowel disease. This elevation of histamine can contribute to the inflammation and tissue damage characteristic of the condition [93]. Additionally, histamine can stimulate the production of other inflammatory mediators, further exacerbating the inflammatory process in Crohn's disease. Certain FAMs, such as *Lentilactobacillus parabuchneri*, *Lentilactobacillus buchneri*, and *Oenococcus oeni* –associated with cheese and wine production– are known histamine producers [94,95]. *O. oeni* is particularly notable for its role in malolactic fermentation [96,97], a process widely used in wine production to reduce acidity and enhance flavor. Meanwhile, *L. parabuchneri* has been detected in numerous cheese varieties, particularly Swiss-type cheese and long-aged cheeses such as Parmigiano Reggiano [98]. *L. buchneri* is similarly found in aged cheeses and is responsible for eye formation in cheeses such as Emmental and Swiss varieties [99]. Interestingly, Swiss-type cheeses with concentrations > 200 mg/kg of histamine are associated with a peppery or burning taste [100]. Fermentation length, product storage, and preparation method can all influence the abundance of histamine at consumption [101]. More research is needed to explore the microbial strain and community dynamics that drive histamine production, as well as how histamine in fermented foods interacts with host biology in both health and disease states, particularly in individuals with inflammatory bowel disease.

Fermented foods can contain other biogenic amines, such as tyramine, a monoamine that may affect blood pressure at excess levels. The enzyme monoamine oxidase in the intestine can break tyramine, and patients taking monoamine oxidase inhibitors (MAOIs) prescribed in the treatment of depression and other disorders should consume fermented foods with caution because little is currently known about the prevalence and concentration of histamine in different fermented foods nor how oral histamine interacts with MAOIs [102].

### Fermented foods and IBS

Fermented foods may offer potential benefits for individuals with IBS, a common gastrointestinal disorder characterized by symptoms such as abdominal pain, bloating, and altered bowel habits. A systematic review [103] found that fermented milk products with probiotic properties may serve as a viable alternative therapy for IBS, although the evidence is still emerging and should be interpreted with caution. Future research should evaluate how high-fiber fermented foods affect IBS subtypes and explore the development of personalized dietary recommendations for these populations. Personalized approaches are key, and individuals with IBS should consider consulting with a healthcare provider or dietitian to identify fermented foods that best support their unique needs and tolerance levels.

For individuals with SIBO, a diet low in fermentable fibers is typically recommended [104]. Food fermentation influences the concentration of fermentable compounds by preprocessing the fibers, which may increase or decrease the concentration of fermentable compounds. Common examples include sourdough and sauerkraut. In sourdough, fermentable oligosaccharides, compounds that may trigger sensitivities in those with SIBO or IBS, decreased following fermentation [105,106]. Conversely, fermenting green cabbage leads to an increase in mannitol [107,108], a sugar alcohol that can act as a trigger for certain patients with IBS.

There is still much to be understood about how FAM and FDM influence the dynamics of potential dietary triggers, such as fermentable fibers, histamine, or sugar alcohols. Key factors include the kinetics of substrate metabolism during fermentation, the role of FAM community structure in producing or degrading these compounds, and how preparation methods—whether at home, in small-batch craft production, or commercial facilities—affect the final product. Additionally, there is a critical need for research to address how these dynamics impact populations with specific health vulnerabilities, such as pregnant individuals and immunocompromised patients, who may have different tolerances or risks associated with fermented food consumption.

## Discussion

The growing body of research on fermented foods highlights their potential to contribute to human health. Although promising, many clinical studies have been limited by factors such as small sample sizes, short durations, and a narrow focus on specific fermented foods or populations. There is a clear need for more rigorous, large-scale trials that address these limitations and explore the impact of different dosages, frequencies, and types of fermented foods on both healthy individuals and diverse patient populations (Table 2) [3,4,47,[109], [110], [111]]. Certain populations, including immunocompromised individuals and those with gastrointestinal conditions, must be cautious in consuming fermented foods, highlighting the importance of personalized nutrition and clinical guidance.

**TABLE 2:** Key focus areas of fermented food clinical research.

Key Focus areas	Current understanding	Gaps in knowledge	Challenges to filling in the gaps	Proposed recommendations	Why is the recommendation valid, given gaps in knowledge?
Consumption of fermented foods	General benefits of fermented food consumption, including gut microbiome diversity and metabolic and immune health.	Defining the impact on health benefit of optimal microbial community, abundance, and impact of FDM, FAM, and probiotics.	Needs might differ among individuals based on stage of life, health status, and baseline microbiome composition.	Encourage patients to include some fermented foods daily.	Our current food supply is mostly devoid of microbes and vastly different than the microbe-laden diet humans evolved with. Fermented foods have been associated with increased gut microbiome diversity and resilience, which are linked to better overall health. Diverse health benefits have been documented. These foods are safe, accessible, and well tolerated.
Establishing serving size	No established serving size for fermented foods. Current servings in clinical trials based on varied definitions.	Serving sizes depend on food type and preparation methods.	Fermented foods are dynamic, making them challenging to study.	Encourage patients to consume tolerable amounts and increase portions gradually.	Evidence of positive effects of fermented food consumption on metabolism [4,109], immune function [3], and mental health [110,111]. See Table 1.
Role of FDMs on health	FDMs include bioactive peptides, vitamins, and organic acids and play a role in altering the nutritional properties, safety, flavor, and texture of the food, as well as shaping microbial community development.	Limited data on dose-response, interaction with individual gut microbiome, and FDM stability.	Metabolite profiles can change over time and storage conditions. Individual differences in genetics and gut microbiota composition could influence how fermentation metabolites are absorbed and metabolized.	Research focused on FDM mechanism, stability, and interaction with host. Patients can choose to eat some fermented products that have been subsequently pasteurized to increase their shelf life, or they can include some fermented products in their cooking.	FDMs, such as short-chain fatty acids, have shown promising effects in reducing the risk of chronic diseases like cardiovascular disease, type 2 diabetes, and even mental health conditions. In addition, human consumption of pasteurized sauerkraut and animal model consumption of sterile-filtered sauerkraut shows similar benefits to consuming food with microbes and without [47,59].
Safety for Special Populations	Immunocompromised and pregnant individuals and those with IBS, SIBO, or histamine intolerance may face unique risks.	Insufficient data on tailored recommendations in these groups.	Variability in FAMs and FDMs complicates safety assessment.	Conduct clinical trials focusing on vulnerable populations and specific needs. Development of stable fermented products tailored to individual populations.	Personalized nutrition can mitigate risk and maximize health benefits for individuals.
Regulatory and Labeling Standards	Marketing terms like “probiotic” and “gut healthy” lack consistency and regulatory clarity.	Ambiguous product claims confuse consumers and healthcare providers.	Regulatory and industry lack of stricter standards, and complexity of FAM and FDM characterization.	Develop clear labeling that specifies whether the product has undergone fermentation, outlining FAM, key FDMs, and pasteurization status.	Transparency in labeling will help both consumers and researchers make informed choices about fermented food product consumption.

*Abbreviations*: FAM, fermentation-associated microbes; FDM, fermentation-derived metabolites; IBS, inflammatory bowel syndrome; SIBO, intestinal bacterial overgrowth.

Although commercial availability of products promoting “gut health,” including fermented foods, has increased, it is essential to differentiate between fermented foods, postbiotic-rich products, and probiotic-supplemented products. Standardized labeling and regulatory oversight will help consumers navigate to products that suit their needs, allowing them to make informed choices and avoid misleading claims. As research continues to expand, elucidating the role fermented foods can play in health, it is likely that available products will further diversify, as will marketing creativity. Transparency and scientific rigor will be key to navigating this evolving landscape and ensuring both public and clinical confidence in the role of fermented foods in health and nutrition.

## Author contributions

The authors’ responsibilities were as follows – EBC and DP: conceptualization; EBC, DP, and CPW: writing–draft; all authors: writing, review, and editing; EBC: visualization; CDG and JLS: supervision; and all authors: read and approved the final manuscript.

## Funding

The authors reported no funding received for this study.

## Conflict of interests

The authors report no conflicts of interest.
